# Dr. Farre's Remonstrance to Mr. Stevenson

**Published:** 1812-12

**Authors:** John Richard Farre

**Affiliations:** Charter-house-square


*To the Editors of the Medical and Physical Journal.*

GENTLEMEN,

A PARAGRAPH at the 257th page of your 164th Number, relating to the editor of the posthumous work of Mr. Saunders, requires a concise reply, which you will oblige me by inserting.

I applaud the "disinterested conviction" of Mr. Stevenson that an improved method of curing cataract, or, let me add, any other disease, ought not to be withheld from the profession at large; and I congratulate him on this liberal change in his sentiments since the 24th of December, 1809. *He is well aware* that Mr. Saunders never listened to the advice of withholding any practical point, especially so important a one as that of enabling surgeons in general to cure cataract—a disease which has hitherto served as the partition wall between the surgeon and the oculist.

I certainly never expected to see the following dubious paragraph, (published, not in the spring of 1812, as its author asserts, but on the 21st of December, 1811, in the Morning Herald,) claimed as a regular notice to the profession of an intended publication.

"The faculty will be gratified to learn, that the *extraordinarily successful mode* of performing the operation for the cataract in children with perfect safety, adopted by the late eminent oculist, Mr. Saunders, and since his death *continually practised by his pupil, Mr. Stevenson*, will not be much longer withheld from the public. Mr. Stevenson is, *we believe*, preparing for the press a practical Treatise on that important subject, feeling it a duty imposed on him by the long delay which has attended the appearance of the posthumous work of Mr. Saunders."

Under any circumstances, I was incapable of being influenced by such a paragraph; but I repel the insinuation that it contributed, in any degree, to the production of the posthumous work of Mr. Saunders, by affirming, that it was not only previously printed off, but also delivered to its very respectable publishers, Messrs. Longman and Co. As its announcement, according to the usual forms of the profession, was exclusively consigned to those gentlemen, *the fact of the completion of the work* was open to any inquirer who had "wishes" on that subject, especially *if he did not expect them to be realised.*

Although, at the period of Mr. Saunders's death (February, 1810), I was engaged in pursuits highly interesting to myself, and which had previously occupied a considerable portion of my time, yet I willingly interrupted them to become the editor of his work. My authority for doing this, was the written request of his widow and brother;—my motive, the desire of offering a tribute to the memory of an intimate friend. I was left at perfect liberty in regard to the time and manner of publication, which was not longer delayed, than my professional duties, and a strict attention to give every fact which could do honor to his name, rendered unavoidable. That Mr. Saunders had left no manuscript account of his operations for the cataract, was not only a matter of the deepest regret, but of serious inconvenience, to me, by compelling me to give a regular detail of the several parts of his inquiry, of which his communication to Mr. Stevenson constitutes but one point. It was this inquiry which appeared to me to be the chief merit of his labors, not that he had suggested this, or adopted that, mode of operating; but that, with a head to devise, and a hand to execute, he was deliberately following out all the possible modes of operating for the cataract, to decide, by a just comparison, their relative advantages.

I am, Gentlemen,

*Charter-house-square,* Your obedient Servant,

*Nov.* 18, 1812. JOHN RICHARD FARRE, M.D.

